# Loss of Arhgap39 facilitates cell migration and invasion in murine hepatocellular cancer cells

**DOI:** 10.32604/or.2024.053791

**Published:** 2025-01-16

**Authors:** HUNG-WEI LIN, PEI YU LEE, YU-SHIUAN CHANG, MAU-SUN CHANG

**Affiliations:** 1 Institute of Biochemical Sciences, National Taiwan University, Taipei, 10617, Taiwan; 2 Institute of Biological Chemistry, Academia Sinica, Taipei, 115201, Taiwan

**Keywords:** Arhgap39, Hepa1-6, Hepa-1c1c7, Invasion

## Abstract

**Background:**

Rho GTPases are essential regulators for cellular movement and intracellular membrane trafficking. Their enzymatic activities fluctuate between active GTP-bound and inactive GDP-bound states regulated by GTPase activating proteins (GAPs) and guanine nucleotide exchange factors (GEFs). Arhgap39/Vilse/Porf-2 is a newly identified GAP. The role of Arhgap39 in migration and invasion has not been addressed thoroughly.

**Methods:**

The Arhgap39 gene was knocked out by Crispr-Cas9 gene editing in mouse Hepa1-6 and Hepa-1c1c7 cells to analyze the impact of Arhgap39 depletion on migration and invasion.

**Results:**

Loss of Arhgap39 noticeably increased the migration and invasive potential. Purified Arhgap39 recombinant protein facilitated the hydrolysis of GTP in RhoA and Rac1 *in vitro*. RNA-seq analysis revealed that matrix metalloproteinase 13 (MMP13) and Laminin subunit beta 1 (LAMB1) were increased in Arhgap39^−/−^ cells. We further crossed Arhgap39fl/fl with KrasLSL-G12D and p53fl/fl mice under the control of albumin-Cre recombinase to induce the spontaneous development of hepatocellular carcinomas. Intriguingly, the expression levels of MMP13 and the overall survival in Alb-Cre_KrasLSL-G12D_p53fl/fl_Arhgap39fl/fl (KPA) mice were comparable to control Alb-Cre_KrasLSL-G12D_p53fl/fl (KP) mice. The cell migration and invasion of KPA mice were also similar to those of control KP mice.

**Conclusion:**

Arhgap39 loss could modulate the migration and invasion in some hepatocellular cancer cells, but not in those isolated from KPA mice.

## Introduction

Rho GTPases belong to the subfamily of Ras small GTPases. They are critical regulators of many cellular activities, such as cell growth, cell movement, intracellular membrane trafficking, and cell-cycle progression [1]. RhoA (Ras homologous member A), Rac1 (Ras-related C3 botulinum toxin substrate 1), and Cdc42 (cell division cycle 42) are most investigated among the Rho family. RhoA is required for stress fiber formation. By contrast, Rac1 and Cdc42 mainly regulate the formation of lamellipodia and filopodia, respectively [2]. Rho GTPases cycle between active GTP-bound and inactive GDP-bound states. Guanine nucleotide exchange factors (GEFs) and GTPase activating proteins (GAPs) are responsible for the switch of GTP- or GDP-bound states. GEFs enhance the exchange of GDP for GTP to activate Rho GTPases [3]. By contrast, GAPs facilitate the intrinsic GTPase activity of Rho GTPases to hydrolyze GTP and turn Rho GTPases off [4]. When Rho GTPases are active, polymerized actin filaments extend the protrusions of lamellipodia and filopodia to invade the surrounding environments and thus facilitate cell migration [5].

Arhgap39/Vilse/Porf-2 is a newly identified RhoGAP, encoding a protein of 1114 amino acid residues and containing three distinctive domains, N-terminal WW (a.a. 65–95), MyTH4 (myosin tail homolog 4) (a.a. 760–875), and C-terminal RhoGAP (a.a. 940–1085) domains [6]. Early studies on Arhgap39 focused on its function in neural development. Arhgap39/Vilse modulates Rac/cdc42-dependent cytoskeletal changes in Drosophila [7,8]. Vilse interacts with the connector enhancer of KSR-2 (CNK2) through the WW domain to regulate Rac cycling during spine morphogenesis in hippocampal neurons [9]. Lee et al. generated the Camk2a-Cre mediated knockout of Arhgap39/Vilse in the forebrain region of mice and found mice with Vilse-deficiency in the hippocampus display reduced electrophysical signals in CA1 hippocampal neurons and exhibit impaired spatial memory [10]. Additionally, Porf-2, a rat Arhgap39 homolog, inhibits the proliferation of neural stem cells (NSCs) via the Wnt/β-Catenin pathway via its RhoGAP domain [11,12]. In rat NSCs, Porf-2 inhibits the activity of the Rac1 and impairs the nuclear translocation of β-catenin, resulting in a repressive proliferation of NSCs [13]. In addition to its function in neural development, Porf-2 reduces cell migration by downregulating the P-2/MMP-9 expression in neuroblastoma and glioma cells [14]. Furthermore, Yao et al. identified Arhgap39 as a biomarker in the immune infiltration of breast cancers [15]. Ding et al. explored the cancer genome atlas (TCGA) database and the result showed that Arhgap39 is highly expressed in hepatocellular carcinoma, possibly leading to a poor prognosis [16].

To further decipher the function of Arhgap39 in tumorigenesis, we knocked out *Arhgap39* by Crispr-Cas9 gene editing in mouse Hepa1-6 and Hepa-1c1c7 cells and revealed the phenotypic alterations due to Arhgap39 deficiency. We further generated spontaneously developing hepatoma in mice to confirm whether the liver cancer cells isolated from *Alb-Cre_Kras*^*LSL-G12D*^*_p53*^*fl/fl*^*_Arhgap39*^*fl/fl*^ (KPA) mice displayed more aggressive migration and invasion than the control cells from *Alb-Cre_Kras*^*LSL-G12D*^*_p53*^*fl/fl*^ (KP) mice.

## Materials and Methods

### Cell lines

Murine hepatocellular Hepa1–6 and Hepa-1c1c7 cells were obtained from American Type Culture Collection (ATCC, Manassas, VA, USA) and cultured in DMEM medium containing 10% fetal bovine serum (FBS) (Hyclone, South Lagan, UT, USA) and 1% penicillin-streptomycin antibiotics (PS) (Hyclone, South Lagan, UT, USA) at 37°C in a CO_2_ incubator (Sanyo, Osaka, Japan). Cells were submitted to mycoplasma detection and short tandem repeats identification for authentication.

### MTT assay

Cells were seeded at a density of 2 × 10^4^ cells onto 96-well plates. Cell viability was measured at the indicated time intervals (0, 24, 48, and 72 h) and verified using the MTT assay (Cayman Chemicals, Ann Arbor, MI, USA). Briefly, cells were added with 10 μL of MTT (5 mg/mL) for 2 h and then incubated with 100 μL of formazan crystal dissolving solution. The resulting purple solution was measured at 570 nm using a microplate reader.

### CRISPR-Cas9 mediated Arhgap39 gene deletion

Two sg-RNA oligos, sgRNA#1 5′-GGCCAGCTTCTCTTGTACAG-3′ and sgRNA#2 5′-CAGACTGGGAGAACTACCAC-3′, were used to knockout Arhgap39 exon 2 containing an ATG translation start site. These two sg-RNAs were independently cloned into the sg-RNA/Cas9 plasmid and co-transfected into Hepa1-6 or Hepa-1c1c7 cells with Lipofectamine 3000 (Invitrogen, Carlsbad, CA, USA) for 72 h. Subsequently, Arhgap39 depleted cells were selected using a serial dilution to isolate a single-cell colony. Selected clones were verified by PCR and immunoblotting to confirm the knockout efficiency.

### Colony formation analysis

1 × 10^3^ cells were seeded on 6-well plates with the DMEM medium and 10% FBS for 12 days. Cells were fixed in 4% paraformaldehyde for 10 min and stained with crystal violet (0.01% in phosphate buffered saline PBS) for 15 min. The colony numbers were scored by ImageJ version 1.47 software (NIH, Bethesda, MD, USA). Three independent experiments were presented as mean ± SD.

### Western blot analysis

Cell extracts were isolated using the RIPA lysis buffer (50 mM Tris HCl, 150 mM NaCl, 1.0% IGEPAL® CA-630, 0.5% Sodium Deoxycholate, 1.0 mM EDTA, 0.1% SDS and 0.01% sodium azide at a pH of 7.4) containing protease inhibitors, and sonicated with 120 W/cm^2^ at 70% amplitude for 5 min using the Sonicator S-4000 (Bioventus, Durham, NC, USA). Protein extracts obtained after centrifugation at 12,000 rpm using a HIMAC centrifuge (Hitachi, Tokyo, Japan) and the protein concentration was determined with the BCA Protein Assay kit (Thermo Scientific, Rockland, IL, USA). Thirty micrograms of protein were subjected to SDS-PAGE. Protein gel was transferred to 0.2 μm of nitrocellulose (NC) membrane (Merck, Burlington, MA, USA) at 100 volts for 90 min. The NC membrane was blotted with 5% non-fat milk in PBS for 2 h at room temperature (RT) and incubated with primary antibody for overnight at 4°C. Subsequently, the NC membrane was washed and incubated with the secondary antibody for 2 h at RT. Finally the immunoblotted bands were developed by SuperSignal West Pico Plus kit (Thermo Scientific, Rockland, IL, USA). Anti-Arhgap39 antibody was described in our previous publication [10]. The sources of other primary antibodies were listed in Table S1. After incubation with the first antibody at 4°C, the membrane was washed twice with Tris-buffered saline with 0.2% Tween-20 (TBST) and then incubated with goat-anti-rabbit or goat-anti-mouse antibodies conjugated with horseradish peroxidase (HRP) for 2 h.

### Wound healing migration analysis

Cells were seeded on 6-well plates and allowed to grow to 90%–95% confluence. Cell wounds were created by a 10 μL of pipette tip and non-adherent cells were removed with 1 × PBS. Cell images were recorded at 0, 18, and 24 h intervals after the creation of wounds under a microscope with a 20 × objective lens (Olympus, Tokyo, Japan). The migration efficiency was determined by calculating the empty areas at different time intervals and was quantified with ImageJ version 1.47 software. Three independent experiments were performed and presented as mean ± SD.

### Cell invasion assay

Invasion chambers were purchased from Corning (Lowell, MA, USA). A mixture of 100 μL of Matrigel (BD Biosciences, Billerica, MA, USA) and 500 μL of serum-free DMEM medium (ratio 1:5) was coated on the upper chamber for 6 h at 37°C. Subsequently, 1 × 10^5^ cells were added to the upper chamber in serum-free DMEM medium, while the lower section contained DMEM medium plus 10% FBS as a chemoattractant. After 48 h incubation, the lower filter was fixed in absolute methanol for 30 min and stained with 0.5% crystal violet for 10 min. Migrated cancer cells were recorded and three independent experiments were carried out and presented as the mean ± SD.

### In vitro RhoGAP assay

GST-tagged Arhgap39 was expressed in insect *SF9* cells and purified with reduced glutathione (Sigma-Aldrich, St. Louis, MI, USA). 0.8 µg/µL of each RhoA, Rac1, and cdc42 proteins were separately mixed with 0.7 µg/µL of purified GST or GST-Arhgap39 accompanied with 200 µM of GTP at room temperature for 20 min. The reaction was terminated by adding 120 µL of CytoPhos reagent (Cytoskeleton, Denver, CO, USA) for 10 min. The released phosphate was determined with the reading of absorbance at 650 nm.

### Phalloidin staining

Cells grown on coverslips were fixed in 4% paraformaldehyde at room temperature for 20 min and then washed twice in 1 × PBS. Cells were added with 0.1% Triton X-100 in 1 × PBS for 5 min to increase permeability and then incubated with rhodamine-phalloidin conjugate at 37°C for 30 min. Coverslips were mounted with mounting medium.

### Next generation sequencing (NGS)

The procedures for the NGS experiment were described previously [17]. Briefly, mRNAs were purified by oligo-T beads (Invitrogen). cDNAs were synthesized, and multiple adapters were ligated to the 5′- and 3′-ends of the cDNAs. A cDNA library was constructed and confirmed on the Agilent 2100 Bio-analyzer (Santa Clara, CA, USA). Subsequently, Illumina NovaSeq sequencing (Illumina, San Diego, CA, USA) was proceeded. Quantification of gene expression was conducted by EBseq. The Heatmap was analyzed through ClustVis (https://biit.cs.ut.ee/clustvis/) (accessed on 25 June 2023). Gene ontology (GO) term (BP, biological process) and Kyoto Encyclopedia of Genes and Genomes (KEGG) pathways were performed using Cluster Profiler.

### Real-time quantitative PCR (RT-qPCR)

Total RNAs were harvested using the TRIzol reagent (Thermo Scientific, Rockland, IL, USA) and the qPCR analysis was performed using 2 × qPCR master mixture (Bioman, Taipei, Taiwan). The thermal program was used: 30 s at 95°C one cycle for denaturation, followed by 40 cycles at 95°C for 15 s, 55°C for 15 s, and 72°C for 20 s. Three independent results were analyzed using 7500 software v.2.0.1 (Applied Biosystems, Foster City, CA, USA) and normalized with glyceraldehyde-3-phosphate dehydrogenase (GAPDH) mRNA. The primer sequences were listed in Table S2.

### Generation of Arhgap39 conditional knockout mice

All animal experiment was approved (approval #13-07-560) by the guidelines of the Institutional Animal Use and Care Committee (Academia Sinica, Taipei, Taiwan). Two males of Alb-Cre mouse at age of 8 weeks (#003574, The Jackson Laboratory, Bar Harbor, ME, USA) was crossed with two females at age 10 weeks of Kras^LSL-G12D^ (#008179, The Jackson Laboratory), two females at age in 10 weeks of p53^fl/fl^ (#008462, The Jackson Laboratory), and two females at age in 10 weeks of PHRF1^fl/fl^ [10] to produce *Alb-Cre_Kras*^*LSL-G12D*^*_p53*^*fl/fl*^, *Alb-Cre_Kras*^*LSL-G12D*^*_p53*^*fl/fl*^*_Arhgap39*^*fl/+*^, and *Alb-Cre_Kras*^*LSL-G12D*^*_p53*^*fl/fl*^*_Arhgap39*^*fl/fl*^ mice. The weights of all animals were between 30–40 g. Mice were housed in standard care, including access to food and water, constant light/dark cycle, and temperature at 25°C. Developing tumors might emerge after 12–16 weeks and cause severe distress or discomfort in mice. If there were over 20% of body weight loss, tumors grown over 10% of body mass, or changes in behaviors, movements, breathing, skin ulcers and abnormal vocalization, animals would be sacrificed in a 100% CO_2_ chamber.

### Genotyping

Mouse tail DNAs were isolated by EasyPure Genomic DNA spin kit (Bioman, Taipei, Taiwan). The following pair of primers were used for Alb-cre, Alb-F1, 5′-GAGCGAGTCTTTCTGCACACAG-3′. CreR1, 5′-TGAGTGAACGAACCTGGTCG-3′. The PCR reaction was conducted by one cycle of 95°C for 8 min followed by 30 cycles of 95°C for 25 s, 55°C for 30 s, and 72°C for 35 s.

### H&E staining

Tissue paraffin sections (4 mm) were deparaffinized in xylene solution and rehydrated through the graded alcohols. Slides were dipped into hematoxylin for 30 s and dipped in 1% eosin Y for 30 s with agitation. Slides were dehydrated in 95% Ethanol for 30 s twice, 100% Ethanol for 30 s, fresh xylene for 30 s, and finally mounted with a mounting medium.

### Primary culture

Primary HCC cells were isolated from tumor masses grown on genetically modified mice with the following procedures. The liver tumors were rinsed once with 1 × PBS and ground into pieces. Collagenase type I in PBS (2 µg/mL) was added and placed in a 37°C incubator (Sanyo, Osaka, Japan) for 2 h with constant shaking. The tissue solution was then incubated with 0.25% trypsin (Gibco, Gaithersburg, MD, USA) for 5 min at 37°C, followed by centrifugation at 300 rpm. Next, the supernatant was gently passed through a 100 µm sterile filter, and the filtrates containing tumor cells were centrifuged (Hitachi, Tokyo, Japan) at 1200 rpm for five minutes. Finally, cell pellets were cultured in DMEM medium with 10% FBS and 1% penicillin/streptomycin.

### Statistical analysis

Statistical calculation was conducted using GraphPad Prism version 7.05 software. All values were expressed as mean ± SD with three independent experiments. The paired Student’s *t*-test was used to show the statistical significance between different groups. *p* < 0.05 was considered statistically significant. *p < 0.05, **p < 0.01, ***p < 0.001.

## Results

### Arhgap39 deficiency increased the colony formation in Hepa1-6 cells

**Figure 1:**
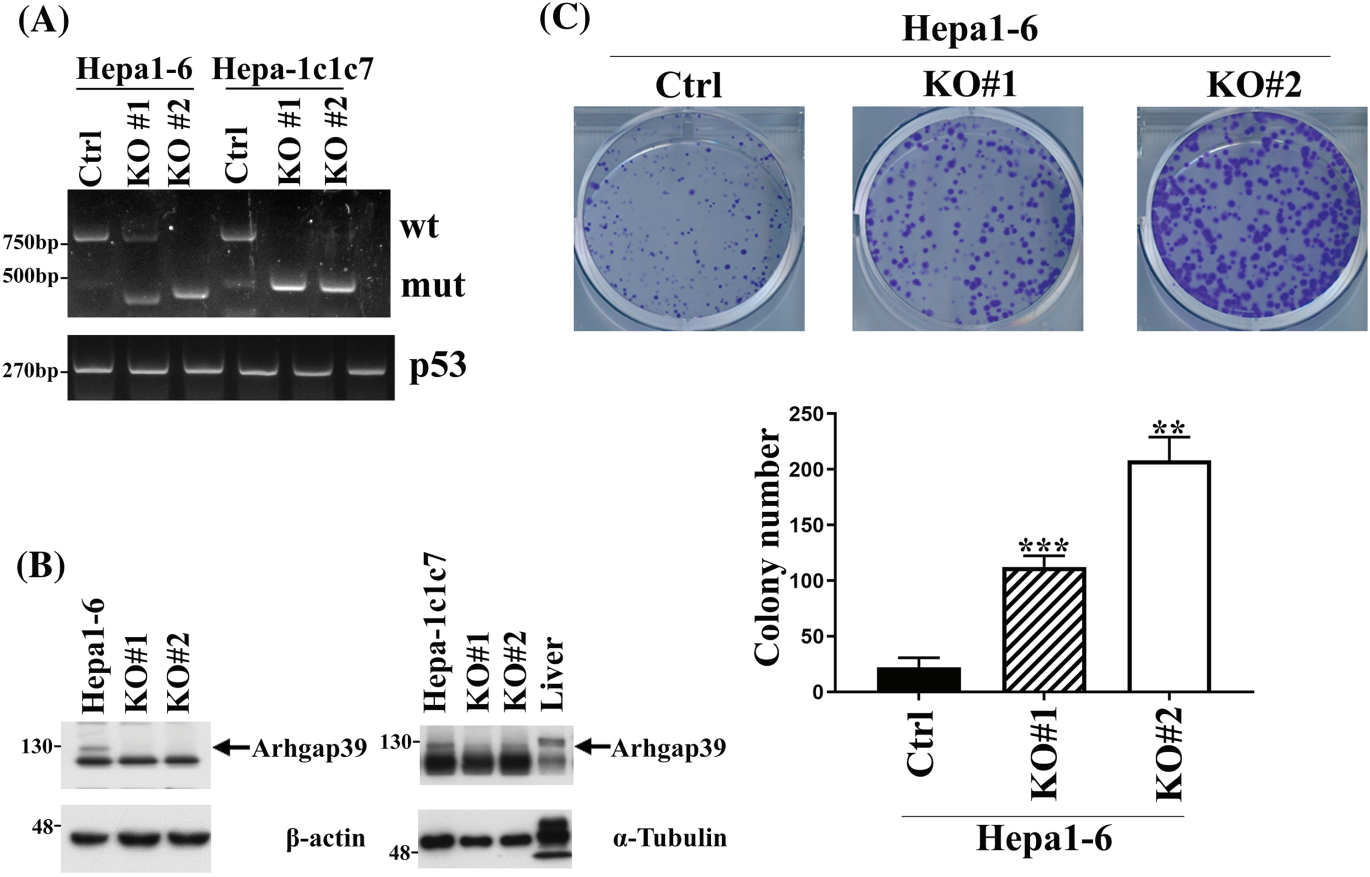
Arhgap39 ablation increased the clonogenic formation in Hepa1-6 and Hepa-1c1c7 cells. (A) Hepa1-6 and Hepa-1c1c7 cells were transfected with two independent Crispr-Cas9 plasmids to knock out the exon 2 of the *Arhgap39* gene. Genotyping PCR was conducted and p53 was used as an internal control. (B) Cells extracts isolated from Arhgap39 KO Hepa1-6 and Hepa1c1c7 cells accompanied with normal mouse liver cells were immunoblotted with indicated antibodies. (C) 1 × 10^3^ Hepa1-6 cells were plated in 6-well dishes for 12 days. Colonies were stained with crystal violet and quantitative results from three independent experiments were analyzed by the Student’s *t*-test. Each bar represents the mean ± SD. **p < 0.01, ***p < 0.001.

To address whether Arhgap39 affected cell survival or cell proliferation, the *Arhgap39* gene was knocked out by Crispr-Cas9 gene editing in murine Hepa1-6 and Hepa-1c1c7 cells. Two different sgRNAs were used to target exon 2 of the *Arhgap39* gene. Genotyping PCR revealed that two different clones exhibited the exon 2 ablation in Hepa1-6 and Hepa-1c1c7 cells (Fig. 1A). Immunoblot analysis showed that the expression of Arhgap39 was lost by Crispr-Cas9 editing (Fig. 1B). Next, we determined whether Arhgap39 ablation affected cell survival. The MTT assay showed that control and Hepa1-6 KO#1 and KO#2 cells displayed similar cell viabilities (Fig. S1A). Additionally, cell growth rates were not significantly different between control and Arhgap39^−/−^ Hepa1-6 KO#1 and KO#2 cells (Fig. S1B). These results indicated that Arhgap39 ablation did not affect short-term cell survival and cell proliferation. By contrast, colony formation experiments revealed that the number of growing colonies in Hepa1-6 KO#1 and KO#2 cells was significantly elevated compared with control Hepa1-6 cells (Fig. 1C), suggesting that the long-term cell proliferation was promoted in the absence of Arhgap39. By contrast, Hepa-1c1c7 and its KO cells were unable to form visible colonies, showing the possibility that cell number and incubation time should be increased or these cells did not exhibit the characteristics of cancer stem cells.

### Arhgap39 depletion increased the cell migration and invasive potential

**Figure 2:**
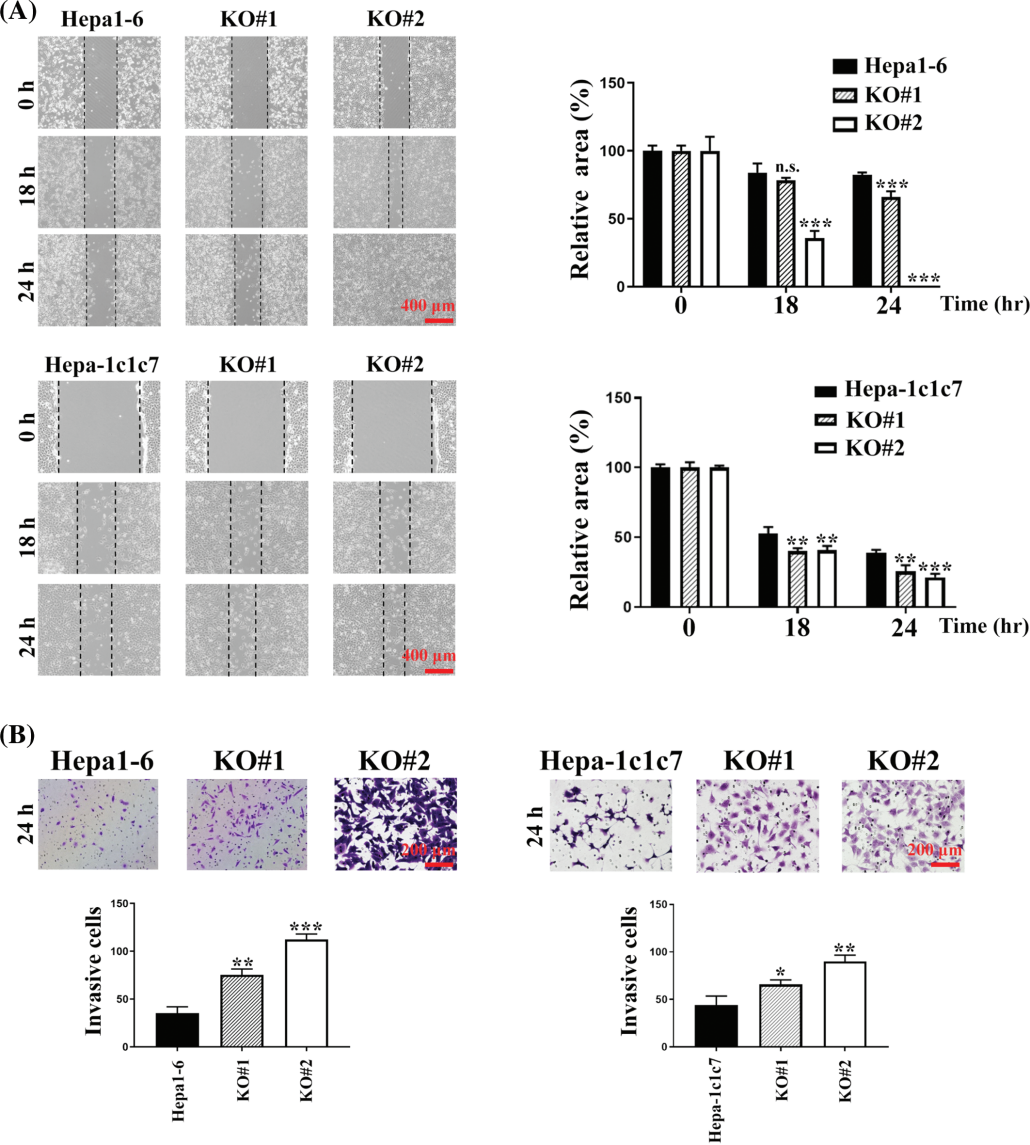
Arhgap39 depletion facilitated cell migration and invasion. (A) Control and Arhgap39 KO Hepa1-6 and Hepa-1c1c7 cells grown on 6-well dishes were scraped with yellow tips and incubated with a complete medium for another 18 h and 24 h. Areas without cells were indicated with dashed lines. Scale bar, 400 μm. (B) Control and Arhgap39 KO Hepa1-6 and Hepa1c1c7 cells were seeded on the Matrigel for the transwell invasion assay for 24 h. Cells migrating to the lower chamber were stained with crystal violet and photographed. Scale bar, 200 nm. The invasion ratio was quantified by calculating cells in the proportional space and analyzed using the Studen’s *t*-tests. Each bar represented the mean ± SD from three independent experiments. n.s., not significant, *p < 0.05, **p < 0.01, ***p < 0.001.

To address the outcome of Arhgap39 deficiency on cell mobility, wound healing and transwell invasion were performed. First, confluent cells were scrapped and incubated with a complete growth medium for 18 h. Arhgap39 ablated Hepa1-6 and Hepa-1c1c7 cells moved much faster than the control cells and almost filled the empty gap after 18 h incubation (Fig. 2A). Next, we performed the transwell invasion in the presence of Matrigel to mimic the extracellular microenvironment. As anticipated, more Arhgap39-deficient cells migrated to the lower membrane compared with control cells (Fig. 2B), suggesting that the absence of Arhgap39 facilitated cellular mobility. Because Hepa1-6 contained more endogenous Arhgap39’s protein (Fig. 1B) and moved faster than Hepa-1c1c7 cells (Fig. 2B), we used Arhgap39 KO (Arhgap39^−/−^) Hepa1-6 cells for the following experiments.

### Arhgap39 inhibited the activity of RhoA and Rac1 in vitro

**Figure 3:**
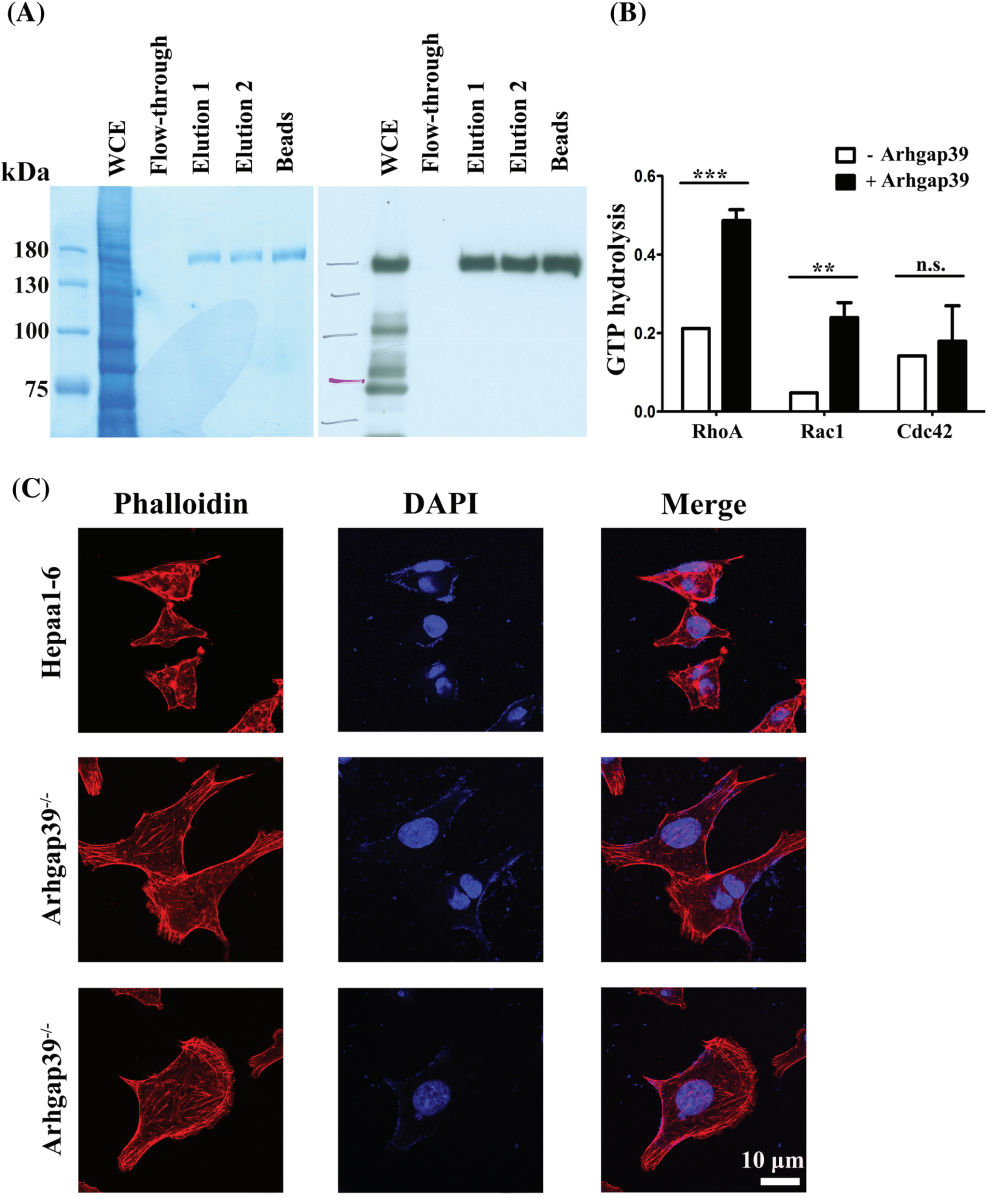
Arhgap39 reduced Rho GTPase activity *in vitro*. (A) GST-tagged Arhgap39 was expressed in insect *SF9* cells and purified with reduced glutathione. The purity of GST-Arhgap39 was determined by Coomassie blue staining (left panel) and immunoblot analysis (right panel). (B) GTP hydrolysis of RhoA, Rac1, and cdc42 protein in the presence of GST or GST-fused Arhgap39 was measured by CytoPhos reagent with the reading of absorbance at 650 nm. (C) Control and Arhgap39^−/−^Hepa1-6 cells were fixed with 4% paraformaldehyde and labeled with phalloidin to detect filamentous actins. Scale Bar, 10 μm. n.s., not significant, **p < 0.01, ***p < 0.001.

To confirm that Arhgap39 would activate the intrinsic activity of Rho GTPase, an *in vitro* Rho GTPase assay was performed. We expressed Glutathione S-transferase (GST)-tagged Arhgap39 in insect *Sf9* cells and purified GST-Arhgap39 proteins by reduced glutathione. Coomassie blue staining and immunoblotting analysis showed the high purity of GST-tagged Arhgap39 (Fig. 3A). Next, GST and GST-tagged Arhgap39 were incubated with GTP-conjugated RhoA, Rac1, and cdc42 to measure the level of hydrolyzed GTP. The results showed that the hydrolysis of GTP to GDP in RhoA and Rac1 was increased in the presence of GST-Arhgap39. By contrast, the intrinsic activity of cdc42 GTPase was slightly affected by Arhgap39 (Fig. 3B), indicating that Arhgap39 would inhibit the activity of RhoA and Rac1. We then stained the filamentous actin with phalloidin in control and Arhgap39^−/−^ Hepa1-6 cells to confirm these results. As expected, most of the Arhgap39^−/−^ Hepa1-6 cells displayed elongated morphology with much higher phalloidin staining. Some cells showed distinctive lamellipodia. By contrast, control Hepa1-6 cells exhibited small and rounded shapes (Fig. 3C), suggesting that Arhgap39 deficiency increases the activity of RhoA and Rac1 and facilitates cell movement.

### Arhgap39 ablation did not increase the expression levels of EMT-related proteins

We speculated that the expression levels of epithelial-mesenchymal transition (EMT) markers might be affected in Arhgap39-depleted cells. However, most EMT markers, snail/zeb1 transcription factors, and E-cadherin and N-cadherin adhesion molecules were largely unchanged (Fig. S2A). We also examined the expression levels of signaling molecules downstream Rho GTPase, such as ROCK1/2 and Arp2/3. The results showed that these Rho GTPase signaling downstream proteins were also largely unchanged (Fig. S2B), suggesting that the absence of Arhgap39 did not affect the expression levels of EMT markers and Rho GTPase signaling to promote cell mobility.

### The altered mRNA profile in Arhgap39^−/−^ Hepa1-6 cell

**Figure 4:**
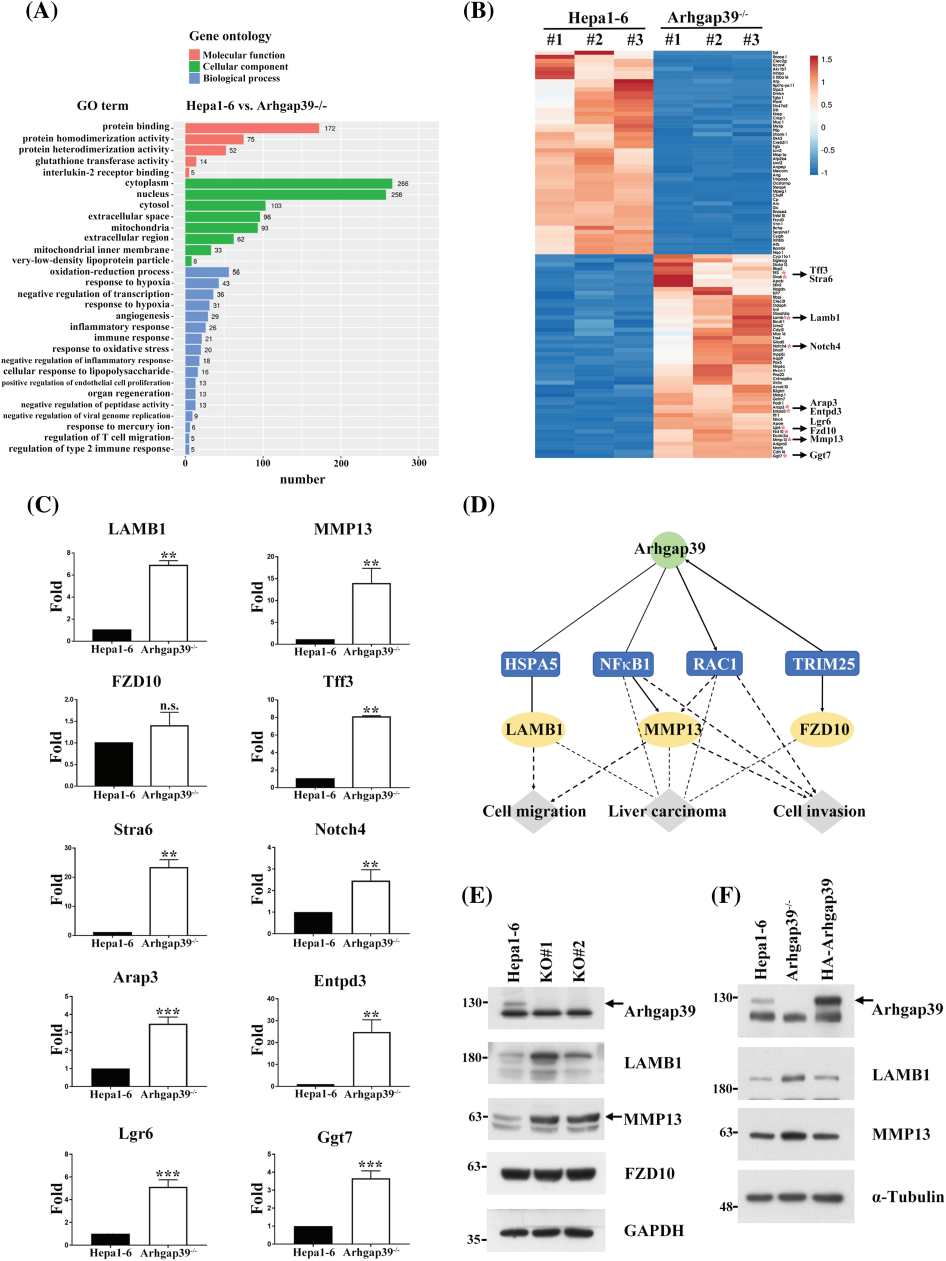
The altered gene profile in Arhgap39^−/−^ Hepa1-6 cells. (A) Gene Ontology analysis displayed the molecular function, cellular components, and biological processes based on the DEGs in Arhgap39^−/−^ Hepa1-6 cells compared with control cells. (B) The heatmap was clustered using ClustVis from the top 50 up-and down-regulated genes of Hepa1-6 and Arhgap39^−/−^ Hepa1-6 cells (n = 3 per group). Down-regulated genes are shown in blue color and up-regulated genes in red color. Ten upregulated genes were labeled with red dots to determine their mRNA contents in the Arhgap39^−/−^ cells. (C) The mRNA levels of ten candidates correlated with liver cancer progression in the top 50 up-regulated genes of Arhgap39^−/−^ group was determined. RT-qPCR results from three independent experiments were normalized to the internal GAPDH and presented as the mean ± SD. n.s., not significant, **p < 0.01, ***p < 0.001 by the Student’s *t*-test. (D) IPA analysis revealed the potential regulatory links between Arhgap39 and MMPs via Rac1 and NF-κB. (E) Cells extracts isolated from control, and Arhgap39-depleted Hepa1-6 cells were blotted with indicated antibodies. (F) Arhgap39^−/−^ Hepa1-6 cells were transfected with HA-Arhgap39, Cell extracts were immunoblotted with indicated antibodies.

To learn the information about the global changes of the transcriptome in Arhgap39^−/−^ Hepa1-6 cells, total RNAs were isolated, and RNA-seq analysis was conducted. Approximately 2080 differentially expressed genes (DEGs) were obtained, in which fold change of mRNA level >2 (up-regulated) or <0.5 (down-regulated) and PPEE < 0.05 are considered statistically significant (Table S3). Gene ontology (GO) analysis revealed that multiple DEGs could be grouped into several distinctive categories of gene ontology. Molecular function (MF) analysis indicates that these genes predominantly engage in protein binding, protein homodimerization, and protein heterodimerization activities. Cellular component (CC) analysis reveals that the co-expressed genes are mainly localized in the cytoplasm, nucleus, and cytosol. Biological process (BP) analysis shows that these genes are primarily involved in oxidation-reduction processes, drug responses, and the negative regulation of transcription from DNA templates (Fig. 4A). We clustered the top fifty up-regulated and down-regulated genes for heatmap analysis (Fig. 4B). Specifically, we selected up-regulated genes on the bottom right for further analysis. These genes had higher expression levels of mRNAs that might promote the migration and invasion in Arhgap39^−/−^ Hepa1-6 cells (Fig. 4B). To confirm RNA-seq results, ten upregulated genes, including LAMB1, MMP13, Fzd10, Notch4, Arpp3, Entpd3, Ggt7, Lgr6, Stra6, and Tff3, were subjected to RT-qPCR analysis. The result indicated that these genes were significantly increased in Arhgap39^−/−^ cells (Fig. 4C). Of note, LAMB1, a carcinoembryonic antigen maker for colorectal cancer, exhibited a 6.95-fold increase. MMP13 showed a 3.91-fold increase in Arhgap39^−/−^ cells (Fig. 4C). To further unravel the complexity of the RNA-seq result, an ingenuity pathways analysis (IPA) was conducted to analyze the correlation of Arhgap39 with the top fifty up-regulated genes. Among multiple potential pathways predicted by the IPA, a promising link between Arhgap39 with Rac1 and NF-κB to regulate MMP13 expression was delineated (Fig. 4D). Subsequently, we examined the protein levels in Arhgap39^−/−^Hepa1-6 cells. Immunoblot analysis showed that the MMP13 and LAMB1 were significantly increased in Arhgap39^−/−^Hepa1-6 cells compared with the control cells (Fig. 4E), echoing the result in the IPA analysis. To confirm whether Arhgap39 modulated the expression of MMP13 and LAMB1 in Arhgap39^−/−^ cells, we transfected HA-Arhgap39 into Arhgap39^−/−^ Hepa1-6 cells. Immunoblotting analysis showed that MMP13 and LAMB1 were reduced in the presence of Arhgap39 (Fig. 4F), supporting that Arhgap39 could affect the expression of MMP13 and LAMB1.

### Generation of tissue-specific knockout of Arhgap39 in the liver

**Figure 5:**
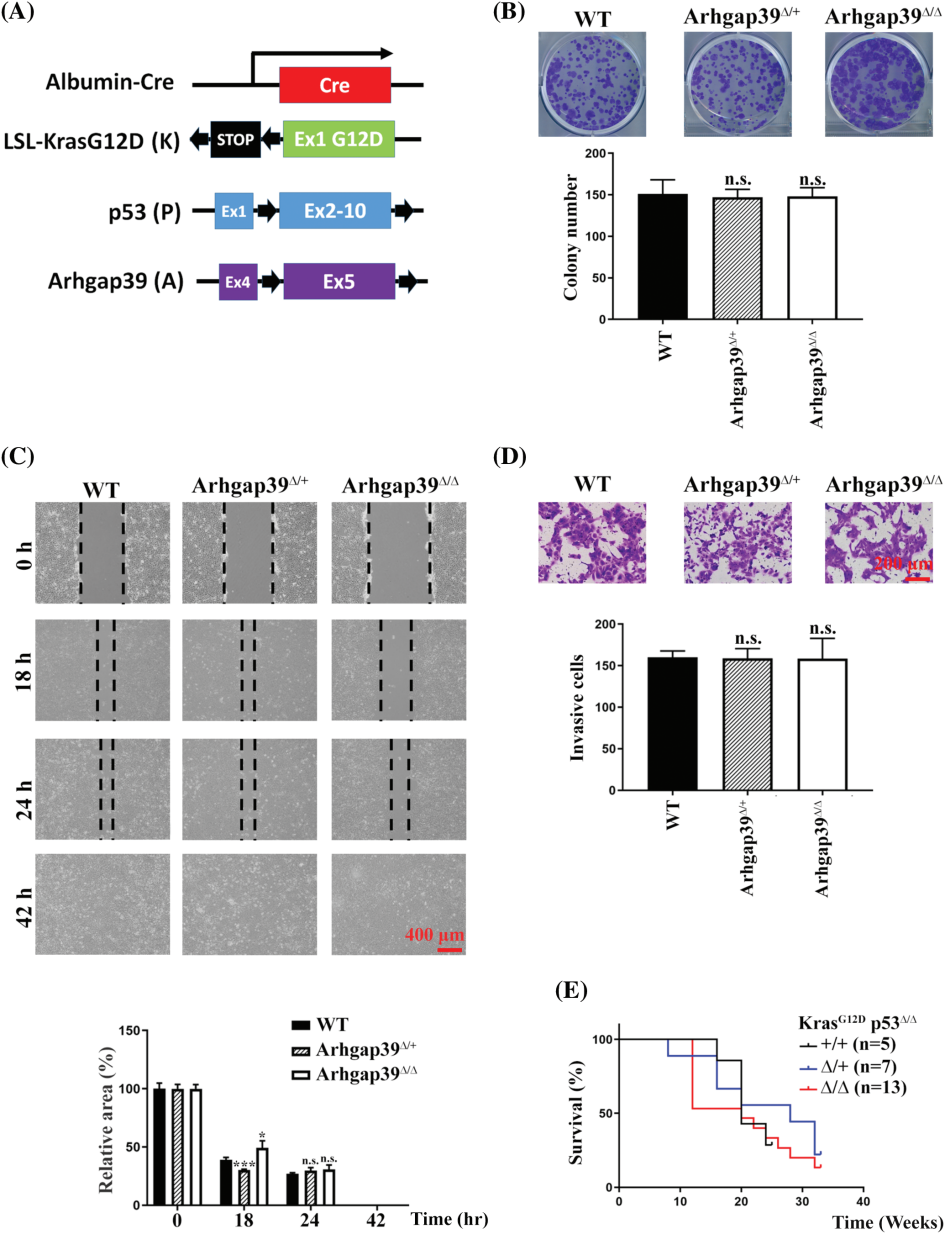
Conditional disruption of *Kras* ^*LSL-G12D*^_ *p53* ^*fl/fl*^ *_Arhgap39* ^*fl/fl*^ genes by Alb-Cre in the liver. (A) Schematic representation of mouse Alb-cre, *LoxP-STOP-LoxP-Kras*^*G12D*^, *p53*, and *Arhgap39* genes. Black arrows indicate the positions of LoxP elements. (B) 1 × 10^3^ cancer cells isolated from KP, KPA^Δ/+^, and KPA^Δ/Δ^ mice were seeded in 6-well dishes for 12 days. Cell colonies were stained with crystal violet. Quantitative results from three independent experiments were analyzed by the Student’s *t*-test. Each bar represents the mean ± SD. n.s., not significant. (C) Wound-healing migration was conducted using primary cancer cells isolated from KP, KPA^Δ/+^, and KPA mice. Scale Bar, 400 μm. n.s., not significant, *p < 0.05, ***p < 0.001. (D) Transwell invasion with matrigel was conducted using primary cancer cells isolated from KP, KPA^Δ/+^, and KPA mice for 24 h. Scale bar, 200 μm. Quantitative results were presented as the mean ± SD by three independent experiments. n.s. not significant by Student’s *t*-test. (E) Kaplan-Meier survival plot of KP (n = 5), KPA^Δ/+^ (n = 7), and KPA^Δ/Δ^ (n = 13) mice.

It is estimated that Kras oncogenic mutation and p53 inactivating mutation occur in 7% and 30% of human HCC cases, respectively [17,18]. To further evaluate the importance of Arhgap39 in tumorigenesis, we crossed *Arhgap39*^*fl/fl*^ mice with *Alb-Cre_Kras*^*G12D*^*_p53*^*fl/fl*^ (KP) mice to produce *Alb-cre_Kras*^*LSL-G12D*^*_p53*^*fl/fl*^*_Arhgap39*^*fl/fl*^ (KPA) and *Alb-Cre_Kras*^*LSL-G12D*^*_p53*^*fl/fl*^*_Arhgap39*^*fl/+*^ (KPA^Δ/+^) mice. Albumin-Cre (Alb-Cre) transgene effectively recombines against floxed alleles in both the adult hepatocytes and cholangiocytes [19]. Both KP and KPA mice carry an oncogenic driver (*Kras*^*G12D*^) and inactivated tumor suppressor (*p53*^*Δ/Δ*^) (Fig. 5A). Using this genetically engineered mouse model, intrahepatic cholangiocarcinomas (iCCAs) and hepatomas will develop as early as ten weeks after birth [20,21]. We anticipated that KPA mice might have a short life span due to the higher migration and invasion caused by the loss of Arhgap39, which in agreement with the results in Hepa1-6 cells.

Genotyping PCR showed that mice carrying different alleles of corresponding mutations were successfully produced (Fig. S3A). KP and KPA mice were both viable with no noticeable developmental abnormalities. As expected, KP and KPA mice gave rise to hepatomas or CCAs ten to twelve weeks after birth. H&E staining of tumor mass did not reveal a significant difference in liver specimens between KP and KPA mice. We dissected the cancer mass from KP and KPA^Δ/Δ^ mice and isolated the cancer cells for cell proliferation and immunoblotting analysis. Immunoblotting results showed that Arhgap39 was genuinely absent in KPA^Δ/Δ^ cancer cells. However, MMP13 and LAMB1 that were elevated in Arhgap39-depleted Hepa1-6 cells remained unchanged between control KP and KPA^Δ/Δ^ cell extracts (Fig. S3B). Cell survival (Fig. S3C) and colony formation (Fig. 5B) data of primary cultured cancer cells were comparable between KP and KPA^Δ/Δ^ mice. Unexpectedly, the wound-healing and invasive potential behaviors were almost identical in cancer cells isolated from KP and KPA^Δ/Δ^ mice (Fig. 5C, D). These results contradicted Figs. 1 and 2 in Hepa1-6 cells. Moreover, the overall survival of KPA mice at clinical endpoints was not statistically different from those of KP and KPA^Δ/+^ mice (Fig. 5E), indicating that some unidentified factors in KPA^Δ/Δ^ animals might compromise the impact of Arhgap39 ablation.

## Discussion

Our data reveal that the absence of Arhgap39 increases the migration and invasion in Arhgap39-depleted cells. RNA-seq analysis and biochemical results showed that increased MMP13 and LAMB1 might potentiate the invasion ability in Hepa1-6 cells. It is interesting to note that the effects of Arhgap39 absence occurred in Hepa1-6 (pERK high expression, pERK^high^) and Hepa-1c1c7 cells (pERK low expression, pERK^low^) cells. Since the aberrations of MEK/ERK signaling substrates play important roles in developing HCC, Arhgap39 might be a novel modulator in several hepatocellular carcinoma cells.

There are twenty members of the Rho GTPase family in humans, but 80 RhoGEFs and 66 are RhoGAPs encoded in the human genome. Most RhoGAPs can inactivate multiple GTPases [4,22]. However, substrate selectivity is largely dependent on the auxiliary domains of RhoGAPs, which are important for controlling the specificity of Rho GTPases [22–24]. Therefore, the liver of KPA mice may express more RhoGAPs than that in Hepa1-6 cells to compensate for the loss of Arhgap39 in tumor progression, which resulted in no difference in the overall survival between control KP and l KP mice. By contrast, the numbers of RhoGAPs expressed in Hepa1-6 and Hepa-1c1c7 cells may be lower than expected and are not sufficient to complement the Arhgap39 deficiency.

Cell migration is important to cancer metastasis [1,2]. During metastasis, tumor cells move from the initial tumor sites into the circulatory system and settle into new niches [3]. Rho GTPases are critical regulatory factors in this metastatic process [4]. The opposing actions of GEFs and GAPs regulate Rho GTPases. Recent studies have suggested that RhoGAPs are typically associated with abnormal activity of Rho proteins during cancer development [25–27]. In this study, the mRNA and protein levels of MMP3 and LAMB1 were increased in Arhgap39-depleted Hepa1-6 cells. MMP13, one of the intestinal collagenases involved in cancer cell invasion [28], has been demonstrated to participate in the migration and metastasis of HCC via TGF-beta pathway [29,30]. LAMB1, also known as Laminin β1, has been found to be highly expressed in invasive gastric tumors [31]. Stabilized LAMB1 by RNA helicase DDX24 promotes hepatocellular carcinoma progression [32]. We found that Arhgap39 deficiency upregulated the expression levels of MMP13 and LAMB1 and reintroduction of Arhgap39 decreased the amounts of MMP13 and LAMB1. Therefore, the migration and invasion of Arhgap39^−/−^ Hepa1-6 cells were highly correlated with the upregulated MMP13 and LAMB1.

The RNA-seq was conducted to analyze the gene expression profile of Arhgap39 ablation in Hepa1-6 cells. DEGs were grouped into three notable patterns of GO, including molecular function, cellular component, and biological process. MMP13 was identified to be involved in the response to drugs and response to hypoxia in the subcategory of biological processes. Additionally, KEGG analysis revealed that the hepatocellular carcinogenesis pathway contained 35 DEGs in the human disease category. LAMB1 was found to be involved in pathways in cancer by KEGG analysis. These findings indicated that MMP13 and LAMB1 represented specific biochemical GO and KEGG pathways by RNA-seq analyses. However, the elevated expression of MMP13 and LAMB1 in Hapa1-6 cells was not found in primary liver cancer cells from KPA mice. Together, these data suggest that the Arhgap39 deficiency alone is unable to promote the invasion capability of KPA mice.

We were wondering whether the absence of Arhgap39 in humans would yield comparable results with Arhgap39^−/−^ Hepa1-6 cells. The association of Arhgap39 expression level with overall survival could be retrieved from the cBioportal TCGA and the Kaplan Meier Plotter datasets. An elevated Arhgap39 expression is associated with low survival in human patients suffering from liver hepatocellular carcinomas and renal clear cell carcinoma. Although these clinical cases do not precisely signify the mutation statuses of *Kras* and *p53*; nonetheless, this result contradicted the overall survival of KPA mice. One plausible explanation is that Arhgap39 is not a critical initiator or driver for tumor progression. Another possibility is that the oncogenic *Kras*^*G12D*^ and inactive *p53* tumor suppressors are so dominant for tumor development that Arhgap39 is unable to impose any deterioration on KPA mice.

However, our study has several limitations. First, the results from KPA mice must be interpreted carefully due to the small sample size. The small sample size might have greater deviation that resulted in no difference between KP and KPA mice. More cases in different cohorts, such as KP and KPA mice, with larger sample sizes are required for validation.

Moreover, the mouse model was not in agreement with the overall survival of human cases retrieved from the cBioportal TCGA and the Kaplan Meier Plotter datasets, In KPA mice, these mice bearing the mutated *Kras*, defective *p53*, and ablated *Arhgap39*. However, there is no specific indication of genetic background in human cases. Additionally, the tumor microenvironment is another essential factor to affects patient survival.

## Conclusion

Our study suggests a potential carcinogenic effect of Arhgap39 in liver cancer progression, especially in Hepa1-6 and Hepa-1c1c7 cells. In our *in vitro* results, we demonstrated that loss of Arhgap39 increases migration and invasion in Arhgap39-depleted cells. We also found that Arhgap39 negatively regulates the migratory ability through modulation of Rho GTPase activity, thereby possibly increasing their metastatic potential. Furthermore, the elevated MMP13 and LAMB1 may further enhance the invasive capabilities in Hepa1-6 cells. The studies on KPA mice with oncogenic *Kras*^*G12D*^, inactive tumor suppressor *p53*, and ablated *Arhgap39* did not further support the importance of Arhgap39 in tumor development. However, Arhgap39 still modulates the invasive potentials in aberrant Hepa1-6 and Hepa-1c1c7 liver cancer cells. Future studies, including determining genotypic signatures in other carcinoma cell lines, investigating the functions of MMP13 and LAMB1 in Arhgap39-depleted cells, and establishing primary culture cells exhibiting oncogenic *Kras*^*G12D*^ and defective *p53*, may ascertain our findings presented in Hepa1-6 and Hepa-1c1c7 cells.

## Supplementary Materials

Figure S1 Arhgap39 ablation did not affect cell viability. **A.** The cell viability of control and Arhgap39-/- Hepa1-6 cells was determined using MTT assay. 1 × 10^4^ cells were seeded per well of 96-well plates in DMEM medium with 10% FBS for 24, 48, and 72 h. **B.** 2 × 10^4^ control and Arhgap39-/- Hepa1-6 cells were incubated DMEM medium with 10% FBS for 24, 48, and 72 h. Cells were calculated and proliferation curves were measured. Each point represented the mean ± SD from three independent experiments. Note that there is no significant difference between control and Arhgap39 KO cells. n.s. not significant, * *p* < 0.05 by Student’s *t*-test.

Figure S2 Arhgap39 depletion did not affect the expression of EMT markers. **A.** Total cell extracts of control, Arhgap39+/-, and Arhgap39-/- Hepa1-6 cells were harvested and immunoblotted with indicated antibodies against EMT-related proteins. **B.** Immunoblotting analysis was conducted using antibodies against representative Rho signaling downstream markers.

Figure S3 Genotyping and colony formation of **A.** Kras^LSL-G12D^, p53^fl/fl^, and Arhgap39 ^fl/fl^ mice were crossed with Alb-Cre transgene mice. The tail DNAs from littermates were isolated for genotyping. The PCR product of the Cre gene was 320 base pairs. The PCR products for wild-type *vs.* mutant of KrasG12D, p53, and Arhgap39 were 250/100, 270/390, and 250/320 base pairs, respectively. **B.** Immunoblotting analysis with indicated antibodies. Note that MMP13 and LAMB1 were unchanged between KPA and KPA^Δ^/^Δ^ mice. C. The cell viability of control and Arhgap39^Δ^/^Δ^ cells was determined using MTT assay. Each point represented the mean ± SD from three independent experiments. Note that there is no significant difference between control and Arhgap39 KO cells. n.s. not significant, **p < 0.01, ***p < 0.001 by Student’s *t*-test.

Figure S4 KEGG pathway enrichment of RNA-seq in Arhgap39-/- Hepa1-6 cells.

Figure S5 Arhgap39 expression is associated with overall survival (OS) of cancer patients. **A.** Hepatocellular carcinoma (left panel, n = 365) and Kidney renal clear cell carcinoma (right panel, n = 512). Patient data were obtained from cBioPortal TCGA PanCancer Atlas datasets. Z score ≥ 2 (red). **B.** Hepatocellular carcinoma (left panel, n = 370) and Kidney renal clear cell carcinoma (right panel, n = 530) were retrieved from the Kaplan–Meier plotter database (http://kmplot.com/analysis/index.php?p=service&cancer). The cohorts were divided into two groups, high (red) and low (black), according to the median expression value of Arhgap39.

## Data Availability

The datasets are available from the corresponding author upon reasonable request.

## Supplementary Materials

The supplementary material is available online at https://doi.org/10.32604/or.2024.053791.
