# Supplementary figures and images for: Loss of Arhgap39 facilitates cell migration and invasion in murine hepatocellular cancer cells
Source: Oncol Res. 2025 Jan 16;33(2):493–503. doi: 10.32604/or.2024.053791 (PMC11753993; doi:10.32604/or.2024.053791)

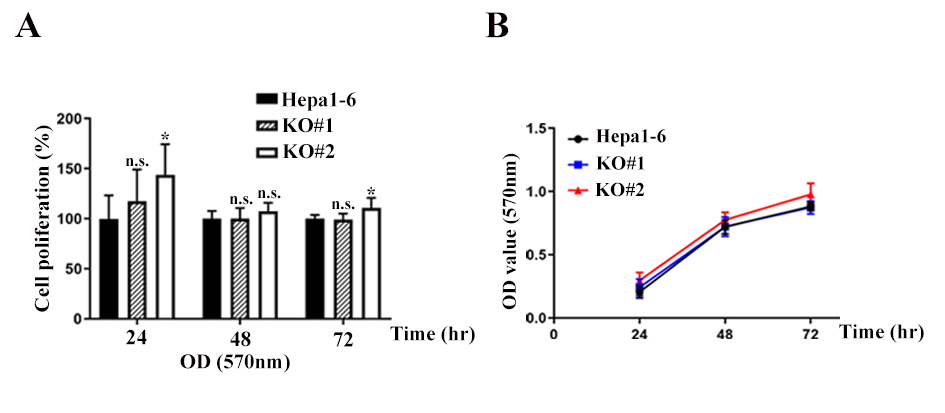

Supplement: Figure S1 — A. The cell viability of control and Arhgap39-/- Hepa1-6 cells was determined using MTT assay. 1 × 10^4^ cells were seeded per well of 96-well plates in DMEM medium with 10% FBS for 24, 48, and 72 h. B. 2 × 10^4^ control and Arhgap39-/- Hepa1-6 cells were incubated DMEM medium with 10% FBS for 24, 48, and 72 h. Cells were calculated and proliferation curves were measured. Each point represented the mean ± SD from three independent experiments. Note that there is no significant difference between control and Arhgap39 KO cells. n.s. not significant, * p < 0.05 by Student’s t-test. [file OncolRes-33-53791-s001.tif]

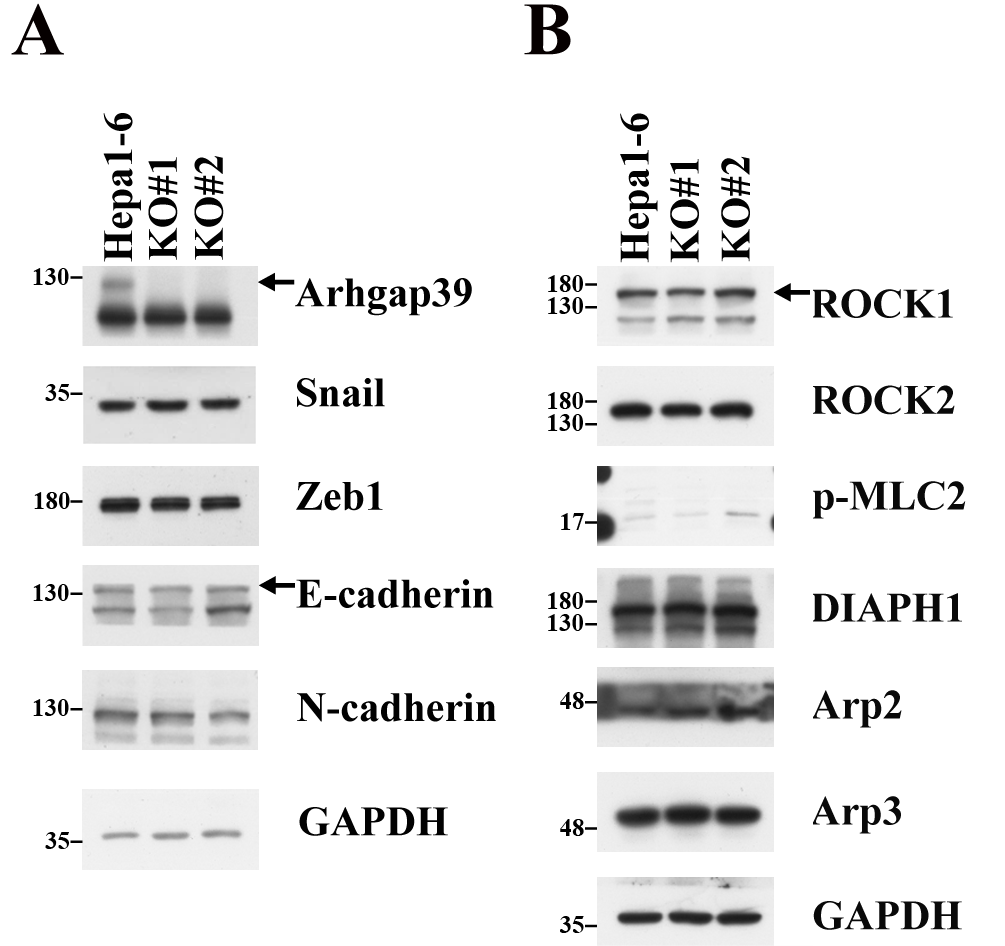

Supplement: Figure S2 — A. Total cell extracts of control, Arhgap39+/-, and Arhgap39-/- Hepa1-6 cells were harvested and immunoblotted with indicated antibodies against EMT-related proteins. B. Immunoblotting analysis was conducted using antibodies against representative Rho signaling downstream markers. [file OncolRes-33-53791-s002.tif]

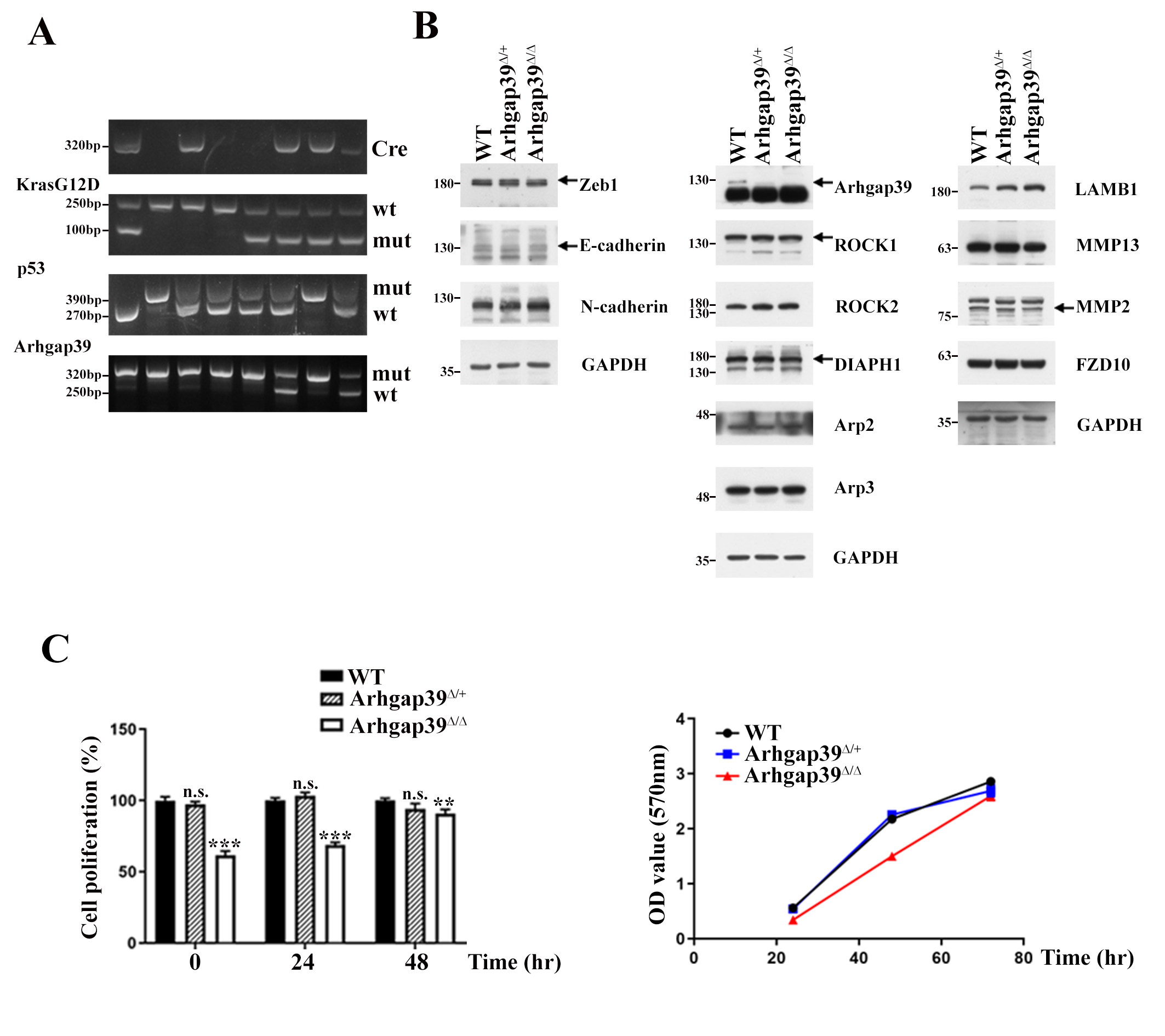

Supplement: Figure S3 [file OncolRes-33-53791-s003.tif]

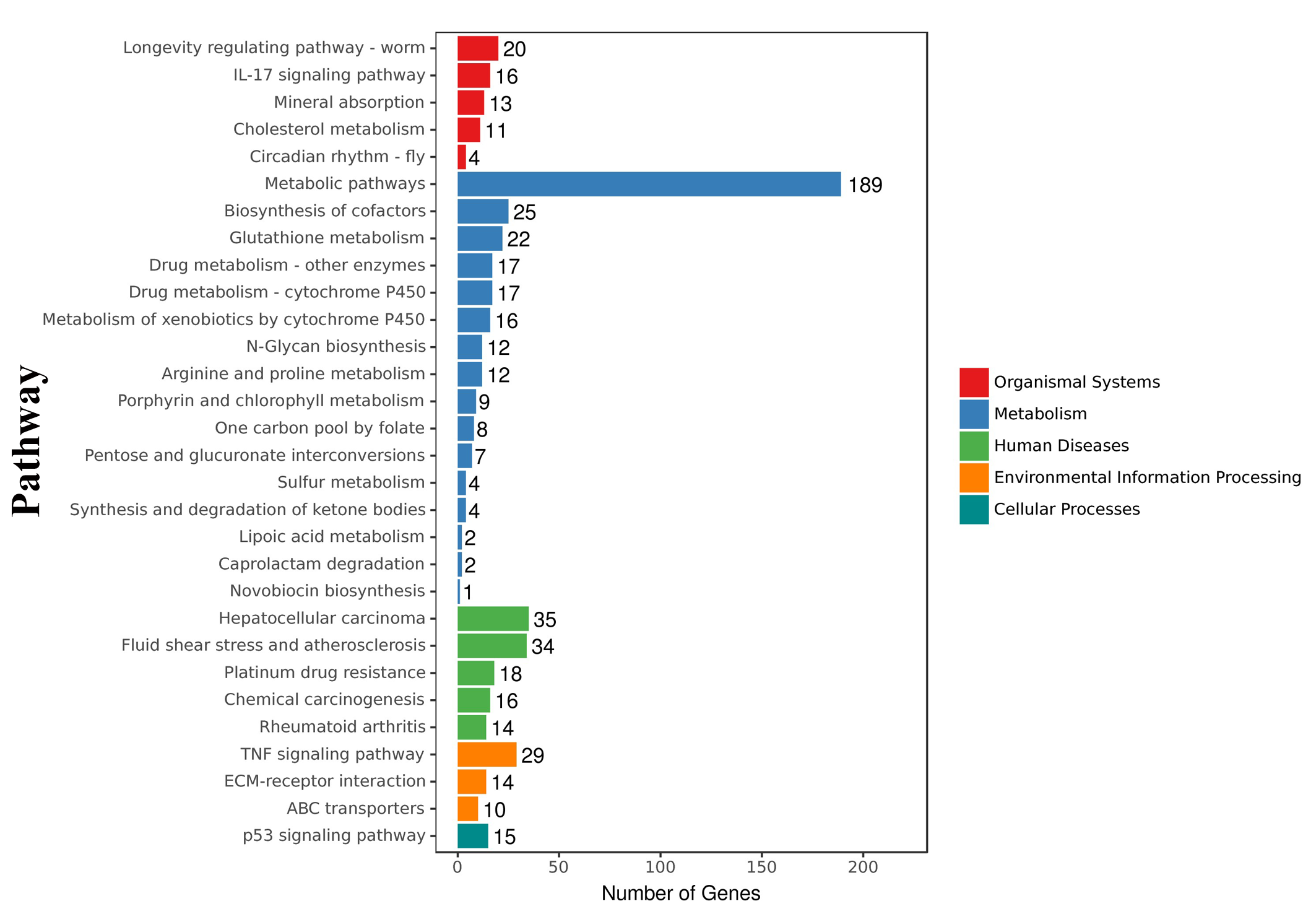

Supplement: Figure S4 [file OncolRes-33-53791-s004.tif]

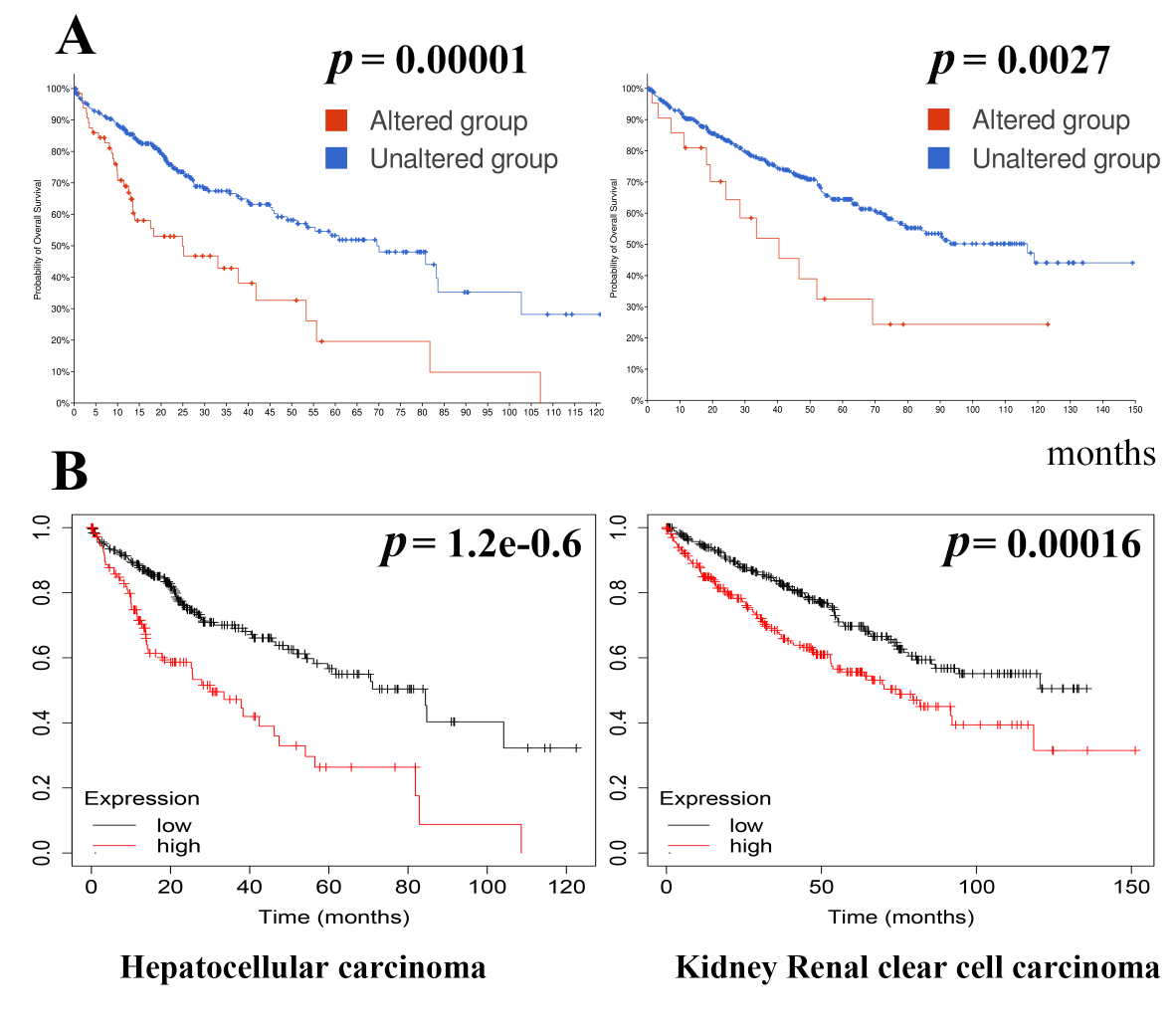

Supplement: Figure S5 — A. Hepatocellular carcinoma (left panel, n = 365) and Kidney renal clear cell carcinoma (right panel, n = 512). Patient data were obtained from cBioPortal TCGA PanCancer Atlas datasets. Z score ≥ 2 (red). B. Hepatocellular carcinoma (left panel, n = 370) and Kidney renal clear cell carcinoma (right panel, n = 530) were retrieved from the Kaplan–Meier plotter database (http://kmplot.com/analysis/index.php?p=service&cancer). The cohorts were divided into two groups, high (red) and low (black), according to the median expression value of Arhgap39. [file OncolRes-33-53791-s005.tif]
