# Supplementary material for: Loss of Arhgap39 facilitates cell migration and invasion in murine hepatocellular cancer cells
Source: Oncol Res. 2025 Jan 16;33(2):493–503. doi: 10.32604/or.2024.053791 (PMC11753993; doi:10.32604/or.2024.053791)
Supplement: Supplementary file 6 [file OncolRes-33-53791-s006.docx]

**Table S1. List of antibodies used in this study**

| Antibody | Company | Catalog number |
| --- | --- | --- |
| β-actin | Sigma | A5441 |
| Snail | Santa Cruz | SC-28199 |
| Twist | Genetex | GTX127310 |
| Zeb1 | Genetex | GTX105278 |
| E-cadherin | Genetex | GTX100443 |
| N-cadherin | Genetex | GTX127345 |
| β-catenin | Genetex | GTX101435 |
| p65(NF-κB) | Cell signaling | #8242S |
| GAPDH | Genetex | GTX100118 |
| Histone H3 | Cell signaling | #9715 |
| PARP1 | Santa Cruz | SC-74470 |
| ROCK1 | Genetex | GTX113266 |
| ROCK2 | Cell signaling | #9029S |
| p-MLC2 | Cell signaling | #3674 |
| DIAPH1 | Genetex | GTX102057 |
| Arp2 | Genetex | GTX103311 |
| Arp3 | Genetex | GTX115345 |
| WAVE1 | Genetex | GTX133263 |
| Fibronectin | Genetex | GTX112794 |
| Lamb1 | Genetex | GTX100787 |
| MMP13 | Genetex | GTX100665 |
| Frizzled10 | Genetex | GTX100358 |

**Table S2. List of qPCR primers used in this study**

| Primer | Forward sequence | Reverse sequence |
| --- | --- | --- |
| Arap3 | 5’-CTACGAGGTGGCTGAGAAGG-3’ | 5’-GACACTCGTGTCCAGCTTCA-3’ |
| Entpd3 | 5’-CCTGTTCCTTTGATGGCATT-3’ | 5’- GTGGGCTGAGAAGCAGTAGG-3’ |
| Ggt7 | 5’-GCAGCAGCAGAGTGTTCTTG-3’ | 5’-TCCACTGAAGAGCCCTGTTT-3’ |
| Lgr6 | 5’-GTGCCAGCTTCTTCAAGACC-3’ | 5’-TTGAGTCCTCTGTCCCCATC-3’ |
| Notch4 | 5’-CCTGTGTGTGGAGTGTACCG-3’ | 5’-GCAATCTTCAGGGTCTCTGC-3’ |
| Lamb1 | 5’-CTGACGAAGGAGAGA-AGAAGTG-3’ | 5’-CTCAGGACATCACGGTCAAAA -3’ |
| MMP13 | 5’-TCTGGGCTCTGAATGGTTATG-3’ | 5’-GCTCAGTCTCTTCACCTCTTT-3’ |
| NLRC4 | 5’-CTCAGAGAACATCCCTGACTATTT-3’ | 5’-CCTGTGACTCTGTAGCTCTTTC -3’ |
| STRA6 | 5’-GCTCTACTATCCAGCCCRCTAT -3’ | 5’-CCAGACCTGAACACCAAAGTG -3’ |
| TFF3 | 5’-CCTGTCTCCAAGCCAATGTAT -3’ | 5’-CAGGGCACATTTGGGATACTG -3’ |
| FZD10 | 5’-CCATGTGCTACTGCGTTTATTC -3’ | 5’-CCAGTCCTTCCTGGATAACATAC -3’ |
| GAPDH | 5’-TCAGTCCCCCACCACACTGAA-3’ | 5’-GATGGTACATGACAAGGTGCG-3’ |
